# Overexpression of Prokineticin 2 in Transgenic Mice Leads to Reduced Circadian Behavioral Rhythmicity and Altered Molecular Rhythms in the Suprachiasmatic Clock

**DOI:** 10.5334/jcr.170

**Published:** 2018-11-06

**Authors:** Xiaohan Li, Chengkang Zhang, Qun-Yong Zhou

**Affiliations:** 1 Department of Pharmacology, University of California, Irvine, CA 92697, US

**Keywords:** prokineticin 2, suprachiasmatic, circadian clock, intrinsically photosensitive retinal ganglion cells, overexpression, output, transgenic mice

## Abstract

In mammals, the master pacemaker driving circadian rhythms is thought to reside in the suprachiasmatic nuclei (SCN) of the anterior hypothalamus. A clear view of molecular clock mechanisms within the SCN neurons has been elucidated. In contrast, much less is known about the output mechanism by which the SCN circadian pacemaker sends timing information for eventual control of physiological and behavioral rhythms. Two secreted molecules, prokineticin 2 (PK2) and vasopressin, that are encoded by respective clock-controlled genes, have been indicated as candidate SCN output molecules. Several lines of evidence have emerged that support the role of PK2 as an output signal for the SCN circadian clock, including the reduced circadian rhythms in mice that are deficient in PK2 or its receptor, PKR2. In the current study, transgenic mice with the overexpression of PK2 have been generated. These transgenic mice displayed reduced oscillation of the PK2 expression in the SCN and decreased amplitude of circadian locomotor rhythm, supporting the important signaling role of PK2 in the regulation of circadian rhythms. Altered molecular rhythms were also observed in the SCN in the transgenic mice, indicating that PK2 signaling also regulates the operation of core clockwork. This conclusion is consistent with recent reports showing the likely signaling role of PK2 from the intrinsically photosensitive retinal ganglion cells to SCN neurons. Thus, PK2 signaling plays roles in both the input and the output pathways of the SCN circadian clock.

## Introduction

Circadian rhythms are the ~24-hour oscillations of the physiological and behavioral processes that are controlled by internal clocks. In mammals, the master pacemaker driving circadian rhythms resides in the suprachiasmatic nuclei (SCN) of the anterior hypothalamus. A clear view of molecular clock mechanisms within the SCN has emerged. The core molecular clockwork consists of autoregulatory transcriptional and translational feedback loops that have both positive and negative elements [27,33]. In contrast, much less is known about the output mechanism by which the SCN circadian pacemaker sends timing information for eventual control of physiological and behavioral rhythms [9,14,15,19,21,23,27,33].

Control of gene transcription is the key to the mechanisms of SCN output. Systematic gene expression profiling has identified 101–337 cycling transcripts in the SCN [24,30], dependent on slightly different criteria and filtering methods used. However, the so-called first-order clock-controlled genes (CCGs), i.e., CCGs that contain E-box enhancers are very rare [24,27,30]. In fact, only nine genes harboring one or more E-box enhancers that are conserved in both human and mouse genomes were identified in these cycling genes [24], illustrating the rarity and also the importance of these first-order CCGs. It is likely that the oscillation of the majority of cycling genes in the SCN is secondary to the oscillation of these first-order CCGs. In mammals, lesion and transplant studies have indicated that signals that mediate the SCN output appear to be secreted molecules [28,29]. Thus, CCGs in the SCN that encode secreted molecules could be crucial signaling molecules for transmitting timing information out of the SCN.

Two secreted molecules, prokineticin 2 (PK2) and vasopressin (AVP), that are encoded by respective CCGs, have been indicated as candidate SCN output molecules [4,12]. AVP is the first CCG identified that regulates circadian rhythm of endocrine and locomotor activities [12,13,14]. Molecular studies have revealed that the transcription of the AVP precursor gene in the SCN is rhythmically regulated by the interaction of core molecular oscillators with E-box enhancers [12]. AVP-deficient Brattleboro rats display attenuated rhythms in a variety of circadian parameters, including body temperature, hormone synthesis, SCN neural firing and sleep-wakefulness [8,11,13,34]. Recent study has indicated that AVP receptors V1a and V1b are critical for mediating AVP in the generation of overt circadian rhythms [18,35].

In vitro and in vivo studies have revealed that PK2 is also a first-order CCG in the SCN, as its mRNA levels are regulated by CLOCK and BMAL1 acting on E-box enhancers in the gene’s promoter [4]. PK2 mRNA exhibits over 50-fold of diurnal oscillation, peaking in the SCN during the middle day and being essentially absent during night [4,5]. This pattern of high amplitude oscillation is altered by light exposure, the main input for entraining and resetting of the SCN clock. The molecular rhythm of PK2 responds asymmetrically to the shifts of light/dark cycles, showing faster adaption to delay than advance of light/dark cycles, consistent with rate of circadian behavioral adaptation [5,36]. Exogenous application of PK2 altered locomotor activity and feeding pattern [4], consistent with PK2 functioning as day time signal. Importantly, PKR2 is densely expressed in essentially all the known primary SCN targets, including the dorsomedial hypothalamic nuclei, paraventricular nuclei of hypothalamus, lateral septum, paraventricular thalamic nuclei and the bed nucleus of the stria terminalis [2,4,6,16,31,32]. Studies using a bacterial artificial chromosome (BAC) transgenic mouse line, in which the enhanced green fluorescence protein (EGFP) reporter gene expression was driven by the PK2 promoter, indicates a circadian oscillation of the number of EGFP-positive neurons in the SCN [38]. The data from this strain of transgenic mice also revealed that EGFP-expressing neurons in the SCN projected to many known SCN target areas, including the ventral lateral septum, medial preoptic area, subparaventricular zone, paraventricular nucleus, dorsomedial hypothalamic nucleus, lateral hypothalamic area and paraventricular thalamic nucleus [38], where the receptor for PK2 (PKR2) is expressed [4,6]. At cellular level, PK2 was shown to regulate the excitability of PKR2-positive neurons, such as the neurons of a primary SCN target, the paraventricular hypothalamic nucleus [37] or the SCN neurons themselves [3,26]. PK2-deficient mice displayed remarkably reduced amplitudes in the oscillation of a variety of circadian parameters, including locomotor activity, sleep–wake cycle, body temperature, circulating glucocorticoid and glucose levels, as well as the expression of peripheral clock genes [10,17]. Deficiency in the PKR2 led to almost identical defects in circadian rhythms as that of PK2-deficiency [25].

In the current study, we provide further supporting genetic evidence for the signaling role of PK2 in the regulation of circadian rhythms by overexpressing PK2 in the transgenic mice.

## Materials and Methods

### Generation of the PKR2-PK2 transgenic mice

A plasmid with the mouse PK2 cDNA under the control of 5.7 kb PKR2 promoter was constructed. The plasmid DNA was linearized and injected into fertilized eggs of wild type mice by microinjection. Founder mice with the incorporation of the plasmid DNA and confirmed PK2 expression at ZT18 in the SCN were identified. PK2 expression in normal mice was essentially undetectable at ZT18 [4]. Ectopic expression of PK2 in non-SCN sites was also detected in PKR2-positive sites. The founder mice were bred with wild type mice to generate the PKR2-PK2 transgenic mice and non-transgenic littermate controls. All animal procedures were approved by the university’s institutional animal care and use committee.

### Measurement and analysis of locomotor circadian rhythms

Monitoring of the circadian locomotor rhythm activity was carried out as described [17,18]. Mice were individually housed with free access to food and water. Mouse cages were equipped with infrared motion sensors for monitoring spontaneous activity and were recorded electronically. Mice were entrained to an initial 12h Light: 12h Dark (LD) cycle (light intensity ~150 lux, lights on at ZT0 7:00 a.m., and lights off at ZT12 7:00 p.m.). After about 2 weeks of activity recording in LD conditions, the mice were placed in constant darkness (DD) with a dim red light (<5 lux) for ~3 weeks. The locomotor activities were calculated as counts per 5-min interval. The free-run period and Fast Fourier Transformation (FFT) were analyzed using ClockLab software (Actimetrics, Evanston, IL) in the MatLab environment. The free-running periods were measured by a χ^2^ periodogram under DD. FFT values representing the peak relative amplitudes in the circadian range were calculated. Statistical analyses were performed by using GraphPad Prism Software Version 5.0 (San Diego, CA).

### In situ hybridization of ^35^S-labeled probe

For the molecular rhythmicity analysis, mutant mice and same number of control mice were sampled every three hours (N = 3 for each time point) under constant darkness for 48 hrs after entrained in 12L:12D for two weeks. *In situ* hybridization was carried out as described [6]. Brains were quickly removed and frozen in isopentane at 20°C for 30 sec. Twenty-micrometer coronal sections were cut on a cryostat and serial sections collected. The following antisense and sense riboprobes were used: PK2 (GenBank accession number AF487280; residues 1–528), mPer1 (340–761 nt of accession number AF022992) or mPer2 (9–489 nt of AF035830), Bmal1 (864–1362 nt of AB015203), mCry1 (1081–1793 nt of AB000777) and mCry2 (981–1765 nt of AB003433). The riboprobes were generated by T7 or SP6 RNA polymerases and radioactively labeled with ^35^S-UTP. Probes were used at concentration of 1 × 10^7^ cpm/ml. Tissue sections were hybridized as follows. Briefly, tissue sections were pretreated with proteinase K, hybridized for 18 hours at 60°C, followed by RNase digestion, high stringency washes and dehydration. Tissue sections were then be exposed to BioMax film (Kodak) for 3–4 days. Specific hybridization signals were quantitatively analyzed by comparing to ^14^C-standards of known radioactivity using a video-based computer image analysis system (MCID, Imaging Research, St. Catharine’s, Ontario, Canada). A calibration curve of optical density versus radioactivity (dpm/mg tissue wet weight) was constructed using ^14^C-standards. Specific hybridization signals in SCN were obtained by subtracting background values obtained from adjacent brain areas that have no hybridization signal. Autoradiographic images were captured using MCID taken under the transillumination microscope (BX50, Olympus) by using Spot camera software version 2.2.2 (Diagnostic Instruments, Sterling Heights, MI).

### In situ hybridization of digoxigenin-labeled probe

Fourteen-micron coronal sections were mounted onto Fisherbrand Superfrost Plus slide (Thermo Fisher Scientific, Pittsburgh, PA). The PK2 cDNA fragment-containing vector was linearized with restriction enzyme and used as template to synthesize anti-sense complementary RNA probes, using a digoxigenin (DIG) RNA labeling mix (Roche Applied Sciences, Indianapolis, IN). *In situ* hybridization and immunological detection of DIG-labeled hybrids was carried out as described previously [38].

## Results

### Generation of the PKR2-PK2 transgenic mice

To investigate the likely effect of perturbed PK2 signaling on circadian rhythms, a plasmid in which the mouse PK2 cDNA under the control of the 5.7 kb PKR2 promoter (PKR2-PK2) was constructed. As PK2 is a secreted molecule that signal through the receptor PKR2 [20], we reasoned expression of PK2 in the PKR2-positive cells would perturb the normal signaling. In vitro study with luciferase reporter gene indicated the activity of this fragment of the PKR2 promoter was active in the cultured cells that express PKR2 endogenously (data not shown). The plasmid DNA was linearized and microinjected into fertilized eggs of CD1 mice to produce the transgenic mice.

**Figure 1:**
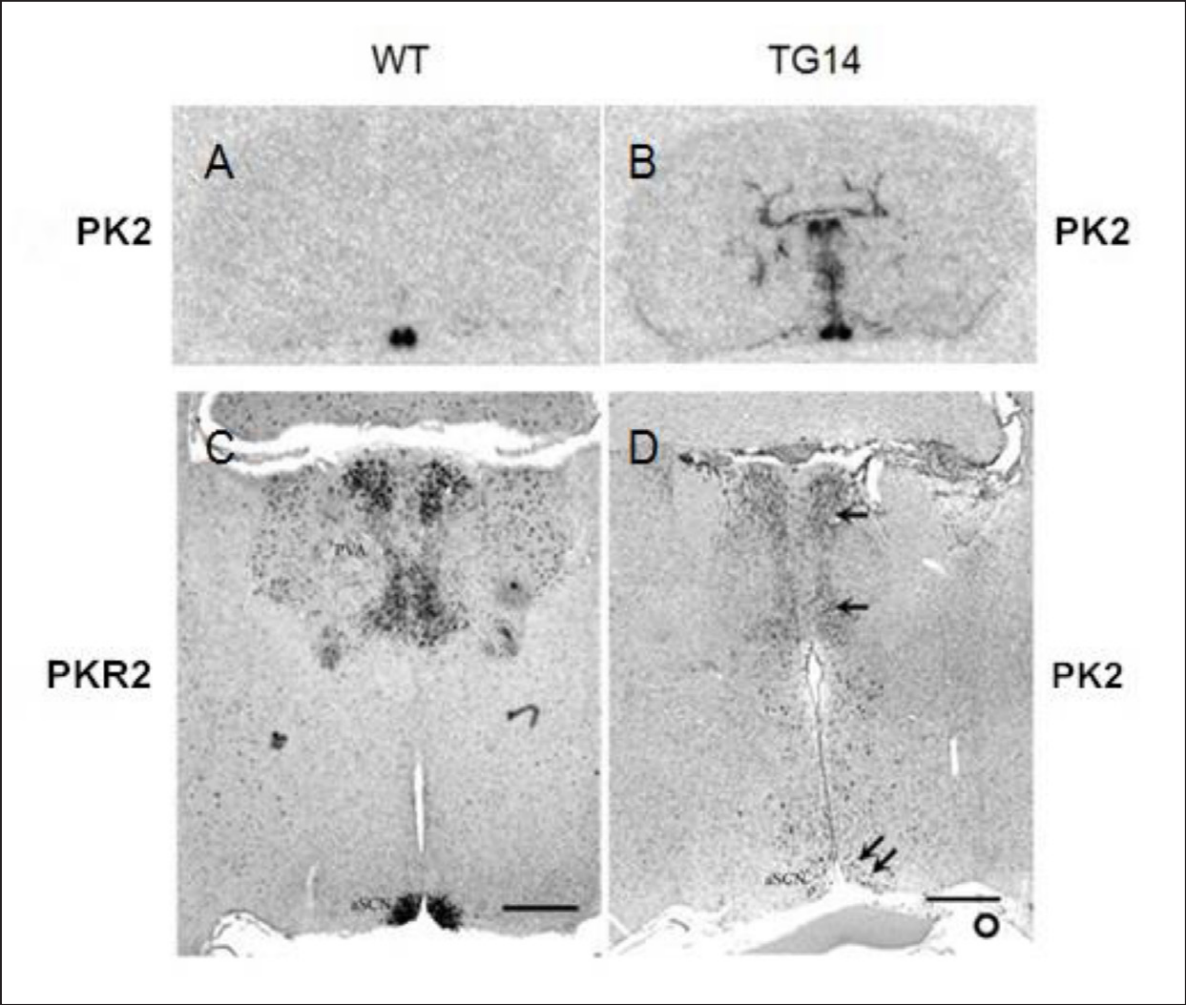
Expression of PK2 in the brain sections of the PKR2-PK2 transgenic mice. PK2 or PKR2 that are labeled at the left and right of the images are the probes used in the *in situ* hybridization. **A** and **B** show the expression of PK2 by *in situ* hybridization with ^35^S probe on brain sections that were sampled at ZT4. Note the selective expression of PK2 in the wild type mice (A) and the ectopic expression of PK2 in transgenic mice, in addition to its expression in the SCN (B). **C** and **D** show the *in situ* hybridization with digoxigenin-labelled probes. The ectopic expression of PK2 in the transgenic mice (single arrow, D) was apparent in the midline thalamus. Such ectopic expression of PK2 correlates with the expression of PKR2 (C). PK2 mRNA was detected in the SCN of the transgenic mouse sampled at ZT18 (double arrow, panel D). At ZT18, only very low level of PK2 expression was found in the SCN of wild type mice. Scale bar, 500 μm.

**Figure 2:**
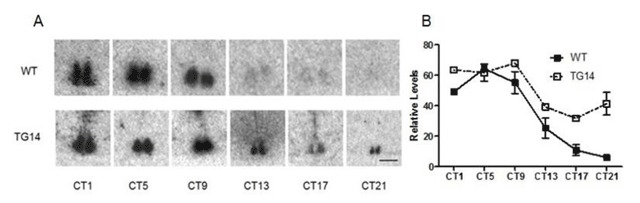
Reduced oscillation of the PK2 expression in the SCN of the PKR2-PK2 transgenic mice. Panel **A** shows the representative images of PK2 mRNA expression in the SCN of the wild type mice (WT) and the PKR2-PK2 transgenic mice (TG14). Panel **B** shows the temporal profiles of quantified PK2 mRNA levels in the SCN. Note the increased PK2 during the subjective dark period, particularly CT17 and CT21, in the transgenic mice. Scale bar, 1 mm.

Two founder mice (TG10 and TG14) with confirmed PK2 expression at ZT 18 in the SCN were identified (Figure 1). PK2 expression in normal mice is essentially undetectable at ZT18 [4]. Also as expected, ectopic PK2 expression was observed in the related brain regions (Figure 1C) that normally express its receptor, PKR2 (Figure 1C), such as the paraventricular nucleus of thalamus. The circadian expression profile of PK2 in the PKR2-PK2 transgenic mice was then examined by sampling every three hours for a continuous 24 hour circle. As shown in Figure 2, the circadian expression profile of PK2 in the SCN of the transgenic mice was altered. Particularly, the amplitude of PK2 oscillation in the SCN of the PKR2-PK2 transgenic mice was significantly reduced, mainly due to the remarkable increase of the PK2 expression during the dark phase (Figure 2).

### Reduced circadian rhythm and behavioral splitting in the PKR2-PK2 transgenic mice

**Figure 3:**
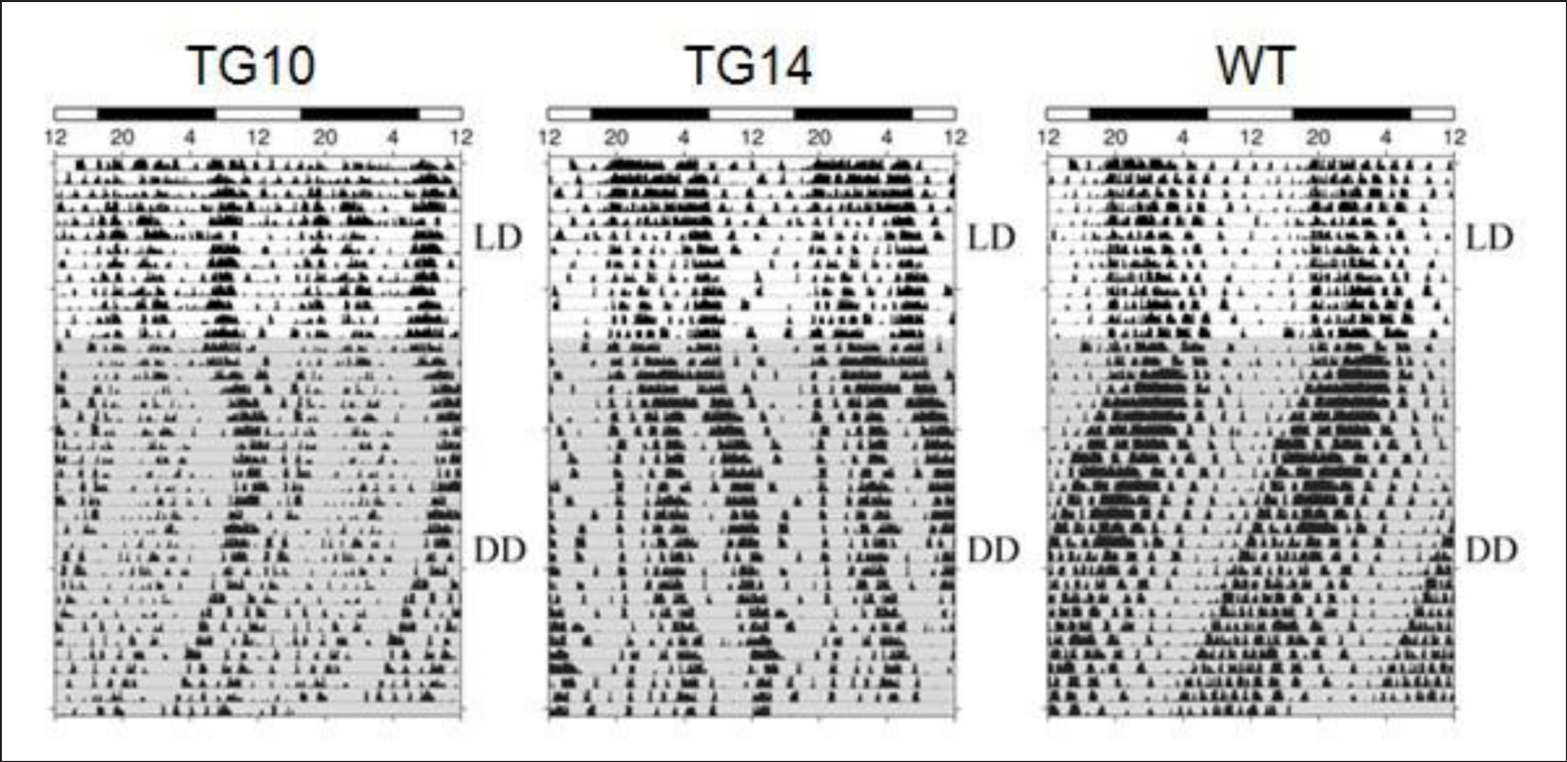
Reduced circadian locomotor rhythmicity in the PKR2-PK2 transgenic mice. The actograms of spontaneous locomotor activities of the PKR2-PK2 transgenic mice and control mice under LD and DD (shadowed) are doubly plotted. Under DD, the transgenic mice displays reduced locomotor rhythmicity. Locomotor rhythm displayed bimodal phenotype, particularly for the mouse line TG14.

**Figure 4:**
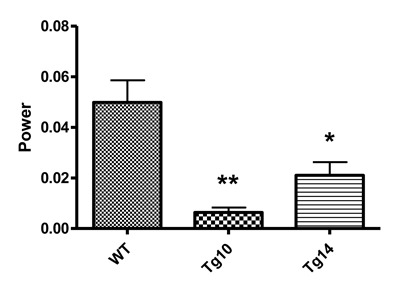
Quantitative analysis of locomotor rhythmicity of the PKR2-PK2 transgenic mice. FFT value, an index for the amplitude of the locomotor circadian rhythmicity was plotted. Both the TG10 and TG14 mice displayed significantly reduced amplitude of circadian rhythmicity. * p < 0.05, ** p < 0.01.

We next investigated the locomotor rhythms of the PKR2-PK2 transgenic mice. The locomotor activities of mice were continuously monitored by using infrared beam recording in their home cages. As illustrated in Figure 3, both lines of the transgenic mice displayed reduced circadian locomotor rhythmicity. Quantification of circadian locomotor rhythmicity revealed that the PKR2-PK2 transgenic mice had significantly lower relative FFT power under DD, compared to their wild type littermate controls (Figure 4). The PKR2-PK2 transgenic mice also appeared to have reduced consolidation in the activity during subject night phase and display apparent behavioral splitting, especially apparent in the TG14 line (Figure 3). In addition, although not statistically significant, the PKR2-PK2 transgenic mice displayed trend of lengthening of the free-running periods (23.43 +/- 0.01 hr for WT, 23.66 +/- 0.27 hr for TG10, 23.98 +/- 0.27 hr for TG14). These studies indicate that overexpression of PK2 under the control of the PKR2 promoter in transgenic mice led to reduced amplitude of circadian locomotor rhythm.

### Altered circadian gene expression in the SCN of the PKR2-PK2 transgenic mice

**Figure 5:**
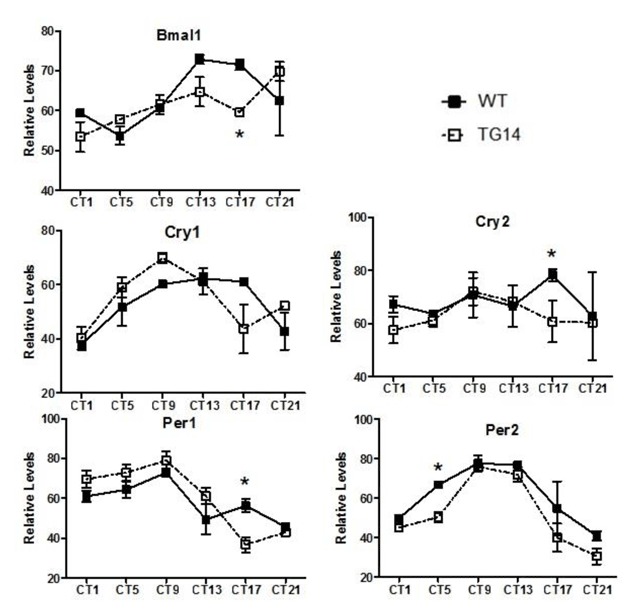
Altered molecular rhythmicity in the SCN of the PKR2-PK2 transgenic mice. The transgenic mice (open square) and wild-type controls (filled squares) were sampled every 3 hr and *in situ* hybridizations on coronal brain sections were performed to quantify the expression of five core oscillator genes. Each data represents mean ± SEM of three mice. * p < 0.05.

We then examined the effects of PK2 overexpression on the rhythm of the SCN circadian genes by *in situ* hybridization (Figure 5). In the SCN of the PKR2-PK2 transgenic mice, the mRNA expression of Bmal1, one of the key core components of the circadian clock, was significantly altered, compared to control mice. The changes in the expression of Cry2, Per1 and Per2 in the SCN of the PKR2-PK2 transgenic mice were also significant, although less remarkable than that of Bmal1 (Figure 5). Subtle alteration of clockwork expression in the SCN and elongated behavioral period had actually previously been noted in PK2-deficient mice [17]. Taken together, these data suggest that PK2 signaling may feedback to affect the expression of the core oscillator genes in the SCN.

## Discussions

### Transgenic overexpression of PK2 leads to reduced oscillation of PK2 mRNA levels in the SCN and decreased amplitude of behavioral rhythm

In this study, we demonstrated that overexpression of PK2 in transgenic mice under the control of its receptor (PKR2) led to reduced oscillation of PK2 mRNA in the SCN and decreased amplitude of circadian locomotor rhythms. The oscillation of PK2 mRNA in the SCN was reduced, primarily due to the increase PK2 expression in the SCN during the dark phase (Figures 1 and 2). The reduction of circadian locomotor rhythm was observed in two lines of independent transgenic mice (Figures 3 and 4). These observations were consistent with the signaling role of PK2 as an output signaling molecule of the SCN circadian clock. PK2 has been postulated as an SCN output molecule based on several previous findings [4,5,17,25,38]. Particularly, results from knockout mice that lack functional PK2 or its receptor (PKR2) have strongly supported the essential role of PK2 signaling for the maintenance of robust circadian rhythms. In the absence of PK2 signaling, the amplitudes of circadian locomotor parameters were remarkably reduced, with rhythmicity amplitude of wheel-running activity of PK2-deficient mice less than 20% of wild type mice [17,25]. Rhythmicity of other circadian parameters, including sleep–wake cycle, body temperature, circulating glucocorticoid and glucose levels, as well as the expression of peripheral clock genes, was also significantly reduced [17,25]. The current overexpression studies in the transgenic mice have provided corroborative evidence for a signaling role of PK2 in the control of circadian rhythms.

### Perturbation of PK2 signaling and molecular rhythms in the SCN

The altered molecular rhythms in the SCN of the PKR2-PK2 transgenic mice indicate that PK2 also appears to regulate the molecular rhythmicity of core clockwork, particularly the amplitude of Bmal 1 (Figure 5). PK2 has been primarily considered as an output signaling molecules of the SCN [4,5,17,25]. However, the receptor for PK2, PKR2, is highly expressed in the SCN [4,6], and SCN neurons are known to form local circuits within the SCN, sending collateral projections within the SCN [1,2,7]. Therefore, in addition to affecting SCN target neurons such as the paraventricular nucleus of thalamus [37], PK2 signaling functions upstream of the SCN, as supported by recent report showing the expression of PK2 in the intrinsically photosensitive retinal ganglion cells that project to SCN neurons [39]. Careful analysis of PK2-deficient mice indicated the free-running periods of residual circadian rhythms in the absence of PK2 signal is slightly lengthened [17]. Subtle alteration in the expression of one clockwork gene was indeed observed in the SCN of PK2-deficient mice [17]. A recent study with mice that have Bmal1 deletion specific to AVP-positive subpopulations of the SCN neurons showed altered circadian expression of genes in the SCN [22], including reduced PK2 expression rhythm. As the PK2 rhythm in the SCN is also reduced in V1a-null mice [18], the two SCN output molecules, PK2 and AVP, may have a similar effect in modulating the molecular rhythm in the SCN. Taken together, these results appear to support the inference that PK2 signaling plays a role in both the input and output of the SCN clock.
